# Phantom Limb Pain in Pediatric Oncology

**DOI:** 10.3389/fneur.2018.00219

**Published:** 2018-04-09

**Authors:** Patrick DeMoss, Logan H. Ramsey, Cynthia Windham Karlson

**Affiliations:** ^1^Department of Pediatrics, University of Mississippi Medical Center, Jackson, MS, United States; ^2^School of Medicine, University of Mississippi Medical Center, Jackson, MS, United States; ^3^Division of Hematology/Oncology, Department of Pediatrics, University of Mississippi Medical Center, Jackson, MS, United States

**Keywords:** phantom limb pain, pediatrics, cancer, therapy, amputation, children, adolescent, treatment

## Abstract

Phantom limb pain (PLP) is a prevalent problem for children and adolescents undergoing amputation due to cancer treatment. The symptoms are wide ranging from sharp to tingling. PLP in children typically lasts for a few minutes but can be almost constant and can be highly distressing. This focused review describes the characteristics, epidemiology, mechanisms, and evidence-based treatment of PLP in pediatric populations, focusing on pediatric cancer. In pediatric oncology, the administration of chemotherapy is a risk factor that potentially sensitizes the nervous system and predisposes pediatric cancer patients to develop PLP after amputation. Gabapentin, tricyclic antidepressants, opiates, nerve blocks, and epidural catheters have shown mixed success in adults and case reports document potential utility in pediatric patients. Non-pharmacologic treatments, such as mirror therapy, psychotherapy, and acupuncture have also been used in pediatric PLP with success. Prospective controlled trials are necessary to advance care for pediatric patients with PLP.

## Introduction

The phenomena of feeling sensation or pain in a limb that is no longer present on the body (phantom limb) was first described hundreds of years ago (1). Phantom limb sensations are defined as non-painful physical sensations perceived to be originating from a missing or amputated body part, most commonly a missing digit or limb (e.g., arm, leg, finger, and toe). Phantom limb sensations are often perceived as kinetic movements, such as toe movement in an amputated foot (2). Phantom limb pain (PLP), by contrast, is the sensation of pain in a missing or amputated body part (2). PLP is noted to reduce quality of life and functional outcomes, as well as be associated with symptoms of depression, thus targeted treatments are needed (3). The present review aims to describe the characteristics, epidemiology, theorized mechanisms, and evidence-based treatment of PLP in pediatric populations, with a focus on pediatric oncology.

## Pediatric PLP Characteristics and Epidemiology

Phantom limb pain is most commonly described as “sharp,” “tingling,” “itching,” “throbbing,” and “stabbing/piercing” (4, 5). Episodes of PLP commonly last seconds to minutes but can be almost constant (4, 6). Episodes typically occur in the afternoon or evening and are more commonly triggered by physical stimuli (6). PLP can occur daily or weekly (4).

In pediatric populations, the most common causes of amputation are trauma, cancer, and congenitally related amputations (4, 5, 7–9). For trauma-related amputations, the prevalence of PLP has been noted between 12 and 83%, with wide variability reported among studies (4, 5, 7–9). For congenitally related amputations, the prevalence of PLP has been noted to be lower, between 3.7 and 20% of children, even occurring in children with congenital absence of a limb (4, 10). In the pediatric oncology population, the prevalence of PLP is also wide ranging between 48 and 90% (5, 7, 11, 12). The onset of pediatric PLP typically occurs shortly after amputation, most often within the first week after amputation (5, 7). Potential gender differences exist between PLP pain triggers in children. In one study, boys reported more physical triggers (e.g., bumping or injuring the amputated limb, walking or sitting for a long time) or were unable to identify a trigger compared to girls who reported more psychosocial triggers (e.g., meeting new people, taking a test, or feeling stressed) (6).

Phantom limb pain may also be related to pre-amputation pain in pediatric populations, although results of this research have been mixed. Pre-amputation pain has been noted to be present in 35–90% of pediatric amputation patients (4, 5, 11). A recent study (2012), however, did not find a significant difference between the experience of pre-amputation pain and the development of PLP post-amputation (11). Similarly, a 1995 study did not find a significant correlation between the experience of pre-amputation pain and post-amputation PLP, but did note the majority of patients experiencing PLP also experienced preoperative pain (5).

For pediatric oncology patients, administration of chemotherapy prior to amputation surgery may increase the risk for PLP or potentially hasten the onset of PLP. Amputation as a means of tumor control is most commonly used in osteosarcoma bone cancer. Additionally, cisplatin and vincristine are chemotherapy agents commonly used to treat osteosarcoma and are both well-known agents for causing peripheral neuropathy (7, 13). In a study examining 67 pediatric oncology patients, 76% of amputees who received chemotherapy before amputation developed PLP within 72 h (7). Consequently, pediatric patients with osteosarcoma appear particularly vulnerable to developing PLP.

The duration of PLP is highly variant. In older studies (1995 and 1998), retrospective surveys have found PLP (of all etiologies) to last years after amputation (4, 5). More recently in 2012 and 2016, only 10–38.9% of pediatric oncology patients reported PLP at 1-year follow-up (11, 12). This difference in duration of PLP is potentially explained by the evolution of preventative and therapeutic measures now available, as discussed in more detail below (11).

## Theorized Mechanisms of PLP

Phantom limb pain is heterogeneous with multiple neurological factors affecting its etiology (14). While the pathophysiology of PLP is complex and mechanisms are not yet understood, the most frequently studied mechanisms of PLP include alterations of the supra-spinal central nervous system (CNS), changes at the level of the spinal cord, and peripheral nerve damage after amputation (15).

Changes in the arrangement of the supra-spinal CNS, including reorganization of the primary motor and primary somatosensory cortices, are the most studied mechanism of PLP (16). It is proposed that functional plasticity of the brain allows adjacent representation zones to extend across the somatosensory cortices and activate nociceptive areas of the missing limb (17, 18). Prior imaging studies have associated proportional intensity of PLP with the magnitude of somatosensory involvement (18). Proposed mechanisms at the spinal cord level include upregulated *N*-methyl-d-aspartate receptors with persistent firing of nociceptive neurons (16, 18, 19).

Proposed mechanisms of PLP within the peripheral nervous system suggest that nerve regeneration and sprouting may drive formation of a neuroma at the amputation site (20). These neuromas are responsible for abnormal afferent impulses to the CNS that then cause the experience of PLP (19, 21). Cell bodies in the dorsal root ganglion are another site of ectopic discharge, with increased sensitivity to sympathetic stimulation and altered expression of sodium channels potentially contributing to the experience of PLP (20).

## Evidence-Based Treatments in Pediatrics

In 2015, the Italian Consensus Conference on Pain on Neurorehabilitation met and reviewed the existent literature to make treatment recommendations for PLP and other neuropathic pain conditions (3). The Italian Consensus Conference on Pain concluded that the scientific evidence for treating PLP is still preliminary, with most support coming from case studies.

### Pharmacologic Treatments

There have been several recent reviews summarizing the use of pharmacologic agents in the management of PLP (2, 22, 23). At this time, no one medication is standard of practice, but several modalities have demonstrated benefits. In pediatric and pediatric oncology reports, medication utilization is similar to adult reports, with most authors reporting some combination of medications for treatment (8, 11, 12).

#### Gabapentin

Gabapentin, a centrally acting anticonvulsant, has been studied for PLP in multiple reviews detailed below. Two adult randomized placebo-controlled trials demonstrated mixed results for the efficacy of gabapentin in reducing PLP, with both studies demonstrating reduced pain intensity but only one study being statistically different than placebo (24, 25). No randomized controlled studies of gabapentin have been performed with pediatric patients; however, several case reports in pediatric cancer document its potential clinical use in isolation or in combination with other therapies (11, 12, 26). In a 2001 case series, gabapentin helped alleviate PLP in three of three pediatric patients at doses 20–35 mg/kg, with pain relief coming suddenly when the therapeutic dose was achieved (26). In a 2012 study of 26 pediatric amputations from cancer, 73% received gabapentin pre-operatively (11). In a 2016 study of 21 pediatric patients with amputations from cancer, the average gabapentin dosage was reported to range from 30.5 to 40.1 mg/kg/day (12). Unfortunately, neither of these studies (2012, 2016) commented on response to gabapentin.

#### Tricyclic Antidepressants

Amitriptyline, a sodium channel blocker used as a tricyclic antidepressant, has also been utilized in pediatric PLP (8, 11, 12, 26, 27) but demonstrates mixed results in adult studies when compared to active placebo (28, 29). As a case series, 10 out of 11 pediatric burn patients who underwent amputation reported improvement of PLP with amitriptyline at doses 25–50 mg (8).

#### Opioids

Opiates are also used in the treatment of PLP. Two studies in adults demonstrated efficacy of a 1-month opiate regimen in treating PLP (29, 30). While the use of opioids has been described for PLP in pediatric cancer patients (8, 11, 12), prescribing opioids for longer than the acute pain phase in pediatric patients with PLP necessitates particular considerations given their potential for abuse.

#### Other Agents

Nerve blocks or epidural catheters have been described in pediatric series (5, 11, 12, 31, 32). Continuous nerve blocks or epidural infusions were used for an average of 5 days in 21 pediatric patients in a 2016 case series (12). Continuous epidural infusions were used post-amputation in 19 pediatric patients in a 2012 case series (11). Unfortunately, the 2012 and 2016 case series were not designed to look at efficacy and did not provide specific medication details. One pediatric series describes the use of ketamine in their patients (amputations related to burns) to reduce PLP; however, the efficacy of ketamine was not reported (8).

### Non-Pharmacologic Treatments in Pediatrics

At this time, no randomized clinical trials of non-pharmacologic treatments have been published for pediatric patients with PLP. The majority of research for non-pharmacologic treatments has focused on mirror therapy in pediatric cancer, while two case reports provide preliminary evidence for complementary modalities such as psychotherapy (33) and acupuncture (34).

#### Mirror Therapy

Mirror therapy provides illusory visual input to a patient by placing a mirror parallel to the healthy limb, generating an intact visual representation of the missing limb. Published randomized controlled trials in adults generally show significant reductions in PLP with the use of mirror therapy (35, 36). For instance, a recent systematic review concluded that 17 of 18 studies demonstrated efficacy of mirror therapy for reducing PLP (36).

Two case reports have described the benefits of mirror therapy in combination with pharmacologic treatment (27) and multimodal rehabilitation (37) for patients with pediatric osteosarcoma bone tumors. In addition, a larger case-control study investigating the efficacy of mirror therapy for PLP in pediatric cancer patients was recently published by Anghelescu and colleagues (12). Researchers conducted a retrospective chart review of 21 children who underwent limb amputation as part of their cancer treatment with 85.7% (*n* = 18) experiencing PLP after amputation. The children treated for PLP with mirror therapy (*n* = 9), in combination with standard care, reported less prevalence (11.1%) and shorter duration (Mean = 246 days) of PLP compared to those who received standard care only (*n* = 9; 66.7% prevalence; Mean duration = 541 days) at 1-year follow-up.

#### Psychotherapy

Psychological therapy may also provide benefit for some patients with PLP. A case report of a 4.5-year-old child who experienced traumatic injury and amputation to the right foot after a motor vehicle accident described successful treatment for PLP using combined psychotherapy and pregabalin (33). This treatment success was observed after initial unsuccessful treatment with paracetamol, ibuprofen, metamizol, morphine, and fentanyl medication management.

#### Acupuncture

In a case report of a 16-year-old female undergoing treatment for osteosarcoma bone tumor, acupuncture was successfully used to reduce PLP and anxiety after leg amputation (34). The adolescent patient completed 12 acupuncture sessions over the course of 6 weeks. Phantom leg pain was reduced from 6/6 to 1/6 at the end of 6 weeks.

## Conclusion

Cancer is one of the primary causes of limb loss in pediatric populations. Children undergoing amputation treatment for osteosarcoma bone tumors often develop PLP, which can persist for months or years after amputation and cause significant distress. While both central and peripheral nervous system mechanisms likely contribute to PLP, children with cancer appear to be at increased risk due to sensitizing factors associated with chemotherapy and pain prior to amputation surgery. The scientific evidence for treating PLP in pediatric populations is still in its infancy, limited by small sample sizes and heterogeneous patient populations. The diverse range of clinical and etiologic presentations creates a unique challenge for researchers and clinicians seeking to advance treatment. More research is needed regarding the pathogenesis of this complex syndrome. Future prospective controlled trials are also needed to more rigorously assess the effectiveness of pharmacologic and non-pharmacologic treatments, in both isolation and combination, for PLP in high-risk pediatric oncology patients. Given that a complete understanding of the disease processes involved in PLP is not currently available, and response to treatment is at least in part individualized, the safest approach to PLP treatment is a combination of pharmacologic and non-pharmacologic modalities. This combination of treatment modalities may be most effective when initiated prior to and immediately following amputation.

## Author Contributions

All authors contributed to the planning and drafting of this manuscript. All authors contributed to the review of research literature and writing of this manuscript. PD and CK contributed to the final editing of the manuscript. All authors are in agreement of the final draft submitted.

## Conflict of Interest Statement

The authors declare that the research was conducted in the absence of any commercial or financial relationships that could be construed as a potential conflict of interest.
